# DNA Sequence Fragment Containing C to A Mutation as a Convenient Mutation Standard for DHPLC Analysis

**Published:** 2013-08

**Authors:** Hassan Dastsooz, Nazanin Vahedi, Majid Fardaei

**Affiliations:** 1 Department of Medical Genetics, Shiraz University of Medical Sciences, Shiraz, Iran

**Keywords:** *ATP7B*, DHPLC, Low range mutation stan- dard

## Abstract

***Objective(s):*** Denaturing high performance liquid chromatography (DHPLC) is a high throughput approach for screening DNA sequence variations. To assess oven calibration, cartridge performance, buffer composition and stability, the WAVE Low and High Range Mutation Standards are employed to ensure reproducibility and accuracy of the chromatographic analysis. The purpose of this study was to provide a cost-effective homemade mutation standard for DHPLC analysis.

***Materials and*** ***Methods:*** DHPLC was performed to evaluate different elution temperatures of a 374 bp DNA fragment with C>A mutation at position of 59 to achieve a peak profile similar to the Low Mutation Standard. In order to verify the reproducibility of the homemade mutation standard using DHPLC, 15 different experiments were performed to compare the homemade mutation standard, the WAVE Low Range Mutation Standard with a positive DNA control sample.

***Results:*** We identified a comparable elution temperature and a peak profile with the WAVE Low Range Mutation Standard.

***Conclusion: ***This study confirmed the reproducibility of the peak profile of our homemade mutation standard compared to the Low Mutation Standard using DHPLC analysis.

## Introduction

In order to detect genetic variants within DNA sequences, many PCR-based techniques are becoming available for the mutation detection. One of the most sensitive and reliable methods to screen mutations is DHPLC analysis (1-3). This method detects DNA variations on the basis of formation of hetero- and homoduplexes between amplified DNA sequences. In order to screen mutations performing this method, having knowledge of the exact location and nature of the mutation is not necessary. Identification of variants in DNA sequences using this method is related to the changes in retention time and peak profiles (1, 4-7). To assess oven calibration, cartridge performance and buffer composition and stability, the WAVE Low and High Range Mutation Standards has been provided by the Transgenomic to ensure that the chromatographic analysis is accurate and reproducible. The WAVE low and high range mutation standards are 209 bp and 219 bp fragments, respectively. These fragments contain a single base pair change. Therefore, performing the DHPLC analysis for the mutation standards gives a specific peak profiles. A change in the peak profile may be due to the changes of cartridge performance, oven calibration and buffer composition. Using high or low range mutation standard is recommended to ensure the stability of the column cartridge (8). The purpose of this study was to compare a homemade mutation standard with a well characterized WAVE Low Mutation Standard.

## Materials and Methods

***DNA samples***

Genomic DNA was extracted from an EDTA-anticoagulated whole blood sample by AccuPrep® Genomic DNA Extraction Kit (Bioneer, Korea) according to the manufacturer's instructions. Informed consent was obtained from all samples before DNA experiment according to the ethical committee of Shiraz University of Medical Sciences. Wild type and DNA fragment containing a point mutation (positive DNA sample) were confirmed by a direct DNA sequencing (Bioneer, Korea) and used for DHPLC analysis. The concentration of Genomic DNA samples were measured by a NanoDrop (ND1000, USA) and stored at -20°C until use.

**Figure 1:**
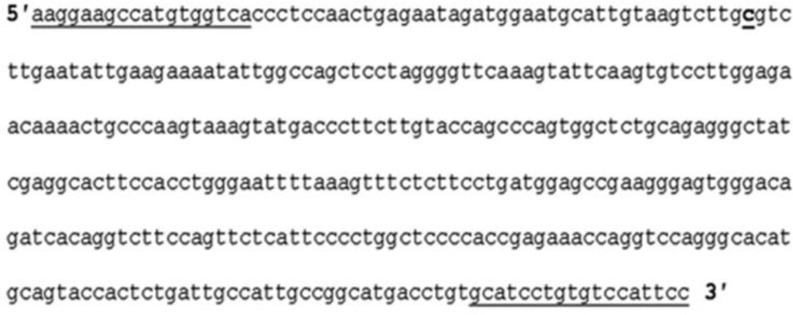
Sequence containing C to A mutation in *ATP7B* gene. Underlined nucleotides are the sequence used for primer pair. Bolded and underlined nucleotide is missense mutation at position 59

***Polymerase chain reaction (PCR)***

A 374 bp fragment in *ATP7B* gene from normal and C to A mutated allele at position 57 (Figure 1) were amplified using primer pair 5′-AAGGAAGCCATGTGG-TCA-3′ and 5′-GGAATGGACACAGGATGC-3′ (9).

**Figure 2:**
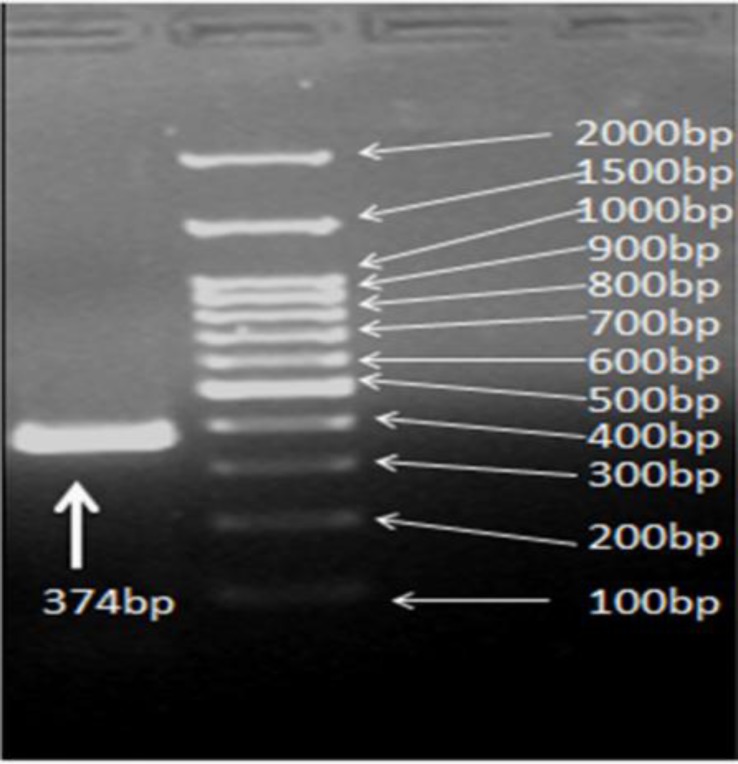
The PCR product from part of exon 2 of *ATP7B* gene seen after gel electrophoresis

Primers 5′-GTGTCGCTCATTGAACTCTC-3′ and 5′-TTCAGAGGAAGTGAGATTTG-3′ (9) were used for the PCR amplifying positive DNA control with a point mutation in *ATP7B* gene. PCR reactions were prepared under the following conditions: 1 µl of each primer (20 pmol/µl), 5 µl Vibuffer A (vivantis, Malaysia), 0.5 µl dNTPs (10 mM), 1.5 µl MgCl2 (50 mM, vivantis), 3 µl DNA template (50-100 ng), 37.8 µl H2O, 0.2 µl Enzyme pfu (5U, vivantis). PCR was performed with a denaturation step at 95°C for 2 min, followed by 35 cycles of annealing temperature step (60°C for analyzed amplicon, homemade mutation standard and 58°C for positive DNA control) for 30 sec, extension step 72°C for 1 min, a further extension step at 72°C for 7 min, and hold at 4°C. The DNA electrophoresis was performed on a 2% agarose gel containing ethidium bromide at voltage of 70 for 45 min and visualized under UV detector (GBOX, SYNGENE, UK), (Figure 2). The PCR products were analyzed directly or stored at -20°C before DHPLC analysis.

**Figure 3:**
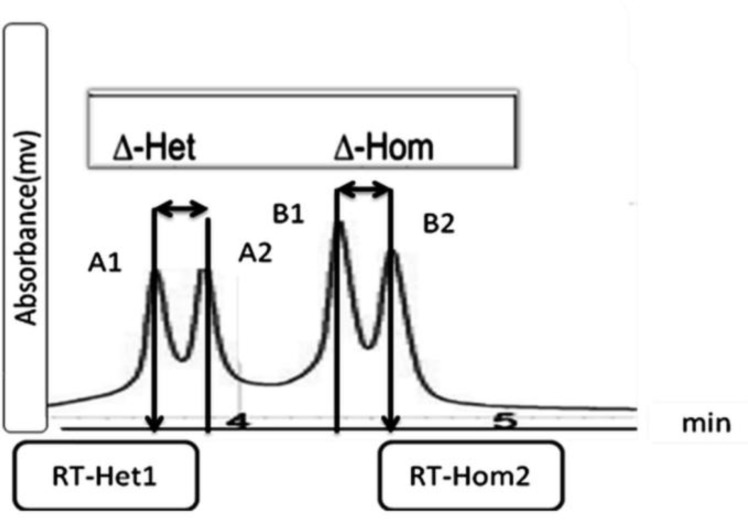
Optimal chromatographic profiles of the WAVE Low Mutation Standard. Retention times (RT) of the Low Mutation Standard are depicted for RT-Het1 (A1), RT-Het2 (A2), RT-Hom1(B1) and RT-Hom2(B2). Delta-Het (∆-Het) and Delta-Hom (∆-Hom) are the difference in RT between the two heteroduplex peaks and the difference in RT between the two homoduplex peaks, respectively

***DHPLC analysis***

Several computer softwares are available to calculate melting profile and melting temperature of the DNA PCR product. In the present study, the Navigator software (Version 3, Transgenomic, USA,) was employed to predict the different elution temperatures for the homemade mutation standard to achieve the best peak profile which were 58, 58.2, and 59.3ºC. The elution temperature for positive DNA control was 61°C.

For heteroduplex formation, equal amount of unpurified PCR products of the analyzed sequence and wild type were mixed and hybridized with denaturing at 95°C for 5 min and slowly cooling to 25°C over 43 min by using Mastercycler gradiant (Hamburg, Germany). Afterwards, eight µl of the products from previous step were automatically loaded into the column and analyzed using buffer B (0.1 M TEAA (Transgenomic, USA) with 25% acetonitrile) and buffer A (0.1 M TEAA, flow rate: 0.90 ml/min).

**Table 1:** The critical parameters for the WAVE Low Range Mutation Standard and DHPLC peak profiles of Homemade Mutation Standard and positive DNA control in 15 experiments

Experiment	RT-Het1 (min)	RT-Hom2 (min)	ΔHet (min)	ΔHom (min)	Peak Intensity (mV) 2 – 12	HMM peak profiles	Positive DNA control peak profile
1	3.653	4.52	0.17	0.19	3.2-4.5	++	++
2	3.68	4.573	0.17	0.19	3-4.2	++	++
3	3.62	4.4	0.17	0.19	4-5.1	++	++
4	3.85	4.6	0.17	0.19	4-5	++	++
5	3.69	4.58	0.17	0.19	3.2-4.2	++	++
6	2.43	2.94	0.07	0.14	4-5	+	+
7	3.65	4.52	0.17	0.19	3.2-4.2	++	++
8	3.687	4.57	0.17	0.19	3.2-4.4	++	++
9	3.80	4.73	0.17	0.19	2.7-4.1	++	++
10	3.72	4.63	0.17	0.19	3-4	++	++
11	3.6	4.527	0.17	0.19	2.5-3.5	++	++
12	3.23	4.25	0.16	0.27	3-4	+	+
13	3.80	4.67	0.17	0.19	3-4.2	++	++
14	3.81	4.58	0.17	0.19	2.9-4.3	++	++
15	3.65	4.5	0.17	0.19	3-4.2	++	++

HMM: homemade mutation standard; ++: very good; +: good

After identification of the best peak profile of the homemade mutation standard, DHPLC analysis was performed using the WAVE system (Transgenomic, USA) for the homemade mutation standard, the Low Range Mutation standard and the positive DNA control in 15 repeated experiments. 

There are some critical parameters to evaluate the reproducibility of the WAVE system using Low Mutation Standard, including detection of all peak profiles with optimal full separation (four peaks, including the 2 heteroduplexes, peaks A1 and A2, and 2 homoduplexes, peaks B1 and B2), the retention time of the first heteroduplex (RT-Het1, normal range is between 3.52 to 4.52 min) and the last homoduplex peaks (RT-Hom2, the normal range is between 4.27 to 5.58 min), the difference in the retention time between the two heteroduplex peaks (Δ-Het, the normal range is between 0.05 to 0.17 min) and the two homoduplex peaks (Δ-Hom, the normal range is between 0.05 to 0.19 min) and peak intensity (the normal range is between 2 to 12 Mv) (Figure 3) (8). 

## Results

**Figure 4:**
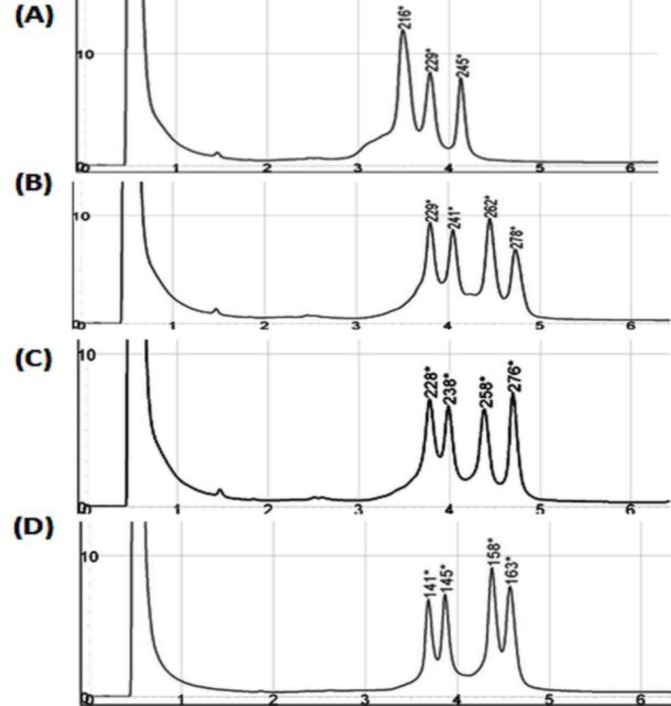
DHPLC peak profiles of the WAVE Low Range Mutation Standard, the homemade mutation standard. (A), (B), and (C) represent DHPLC peak profiles of the homemade mutation standard at 59.3°C, 58°C, and 58.2°C, respectively. (D) DHPLC peak profile of the Low Mutation Standard at 56°C which showed the beast conditions for the WAVE system in this study

Separation of heteroduplex formed products was analyzed at different temperatures. Comparing to Low Mutation Standard, the same quality of the peak profile was generated for homemade mutation standard at 58.2°C. The DHPLC peak profiles of homemade mutation standard at different elution temperatures and Low Mutation Standard are illustrated in Figure 4.

In 13 of 15 repeated experiments, we have observed that the critical parameters for the WAVE Low Mutation Standard were in a normal range. Consequently the homemade mutation standard and positive DNA control showed the best peak profiles. In two experiments (6 and 12), we have observed the little diversion from a normal range for the Low Mutation Standard, therefore, the homemade mutation standard and the positive DNA control showed weak DHPLC peaks profiles (Table 1). In general, results of the present study showed reproducibility of this homemade mutation standard. 

## Discussion

Analytical separation in the WAVE System is on the basis of reverse-phase ion-pair liquid chromatography which is performed under conditions where temperature may be changed automatically. Two essential steps allow detecting variations in DNA sequence, including the interaction of negative charges of DNA backbone with the DNASep® cartridge matrix which is performed by the ion-pair reagent 0.1 M triethylammonium acetate (TEAA) and elution of bounded DNA from the cartridge matrix by an organic mobile phase, 0.1 M triethylammonium acetate in 25% acetonitrile. To detect mutations and SNPs by DHPLC analysis, partially denaturing conditions are generated by heating the oven to a temperature between 50 and 70°C (8, 10, 11).

In theory, for any nucleotide changes in DNA sequence, two double peaks are detectable in case of optimal conditions. They include the first double peak and the second double peak for the two heteroduplexes and the two homoduplexes, respectively. The lower hydrophobicity of heteroduplexes results in its earlier elution in comparison to homoduplexes. It is worth noting that in practice, different peak numbers can be detected. The presence of different numbers of heteroduplex and homoduplex peaks is dependent on several factors include the length and sequence of the DNA fragment which determines melting temperature of the analyzed DNA sequence, hydrogen bonding between non Watson-Crick base oppositions, the presence of the specific sequence change and the melting characteristics of the surrounding nucleotides of a mutation or SNP (12-15). 

According to the data from DHPLC analysis of the wave mutation standards, homemade mutation standard and positive DNA control, we have suggested that using a positive DNA control accompanying by a homemade mutation standard may be very useful to verify the correct function of the wave system and buffer composition.

## Conclusion

A homemade mutation standard was provided for DHPLC. Therefore, we have suggested that the fragment with these characteristic peaks profiles can be used as a convenient mutation standard for the WAVE nucleic acid fragment analysis system.

## References

[1] Guida V, Colosimo A, Fiorito M, Foglietta E, Bianco I, Ivaldi G (2004). Denaturing HPLC-based assay for molecular screening of nondeletional mutations causing alpha-thalassemias. Clin Chem.

[2] Lin D, Goldstein JA, Mhatre AN, Lustig LR, Pfister M, Lalwani AK (2001). Assessment of denaturing high-performance liquid chromatography (DHPLC) in screening for mutations in connexin 26 (GJB2). Hum Mutat.

[3] van Den Bosch BJ, de Coo RF, Scholte HR, Nijland JG, van Den Bogaard R, de Visser M (2000). Mutation analysis of the entire mitochondrial genome using denaturing high performance liquid chromatography. Nucleic Acids Res.

[4] Kuklin A, Munson K, Gjerde D, Haefele R, Taylor P (1997). Detection of single-nucleotide polymorphisms with the WAVE DNA fragment analysis system. Genet Test.

[5] Larsen LA, Christiansen M, Vuust J, Andersen PS (2001). Recent developments in high-throughput mutation screening. Pharmacogenomics.

[6] Wagner T, Stoppa-Lyonnet D, Fleischmann E, Muhr D, Pages S, Sandberg T (1999). Denaturing high-performance liquid chromatography detects reliably BRCA1 and BRCA2 mutations. Genomics.

[7] Yu B, Sawyer NA, Chiu C, Oefner PJ, Underhill PA (2006). DNA mutation detection using denaturing high-performance liquid chromatography (DHPLC). Curr Protoc Hum Genet.

[8] Schollen E, Dequeker E, McQuaid S, Vankeirsbilck B, Michils G, Harvey J (2005). Diagnostic DHPLC Quality Assurance (DDQA): a collaborative approach to the generation of validated and standardized methods for DHPLC-based mutation screening in clinical genetics laboratories. Hum Mutat.

[9] Panichareon B, Taweechue K, Thongnoppakhun W, Aksornworanart M, Pithukpakorn M, Yenchitsomanus PT (2011). Six novel ATP7B mutations in Thai patients with Wilson disease. Eur J Med Genet.

[10] O'Donovan MC, Oefner PJ, Roberts SC, Austin J, Hoogendoorn B, Guy C (1998). Blind analysis of denaturing high-performance liquid chromatography as a tool for mutation detection. Genomics.

[11] Gross E, Arnold N, Pfeifer K, Bandick K, Kiechle M (2000). Identification of specific BRCA1 and BRCA2 variants by DHPLC. Hum Mutat.

[12] Ke SH, Wartell RM (1993). Influence of nearest neighbor sequence on the stability of base pair mismatches in long DNA; determination by temperature-gradient gel electrophoresis. Nucleic Acids Res.

[13] Oldenburg J, Ivaskevicius V, Rost S, Fregin A, White K, Holinski-Feder E (2001). Evaluation of DHPLC in the analysis of hemophilia A. J Biochem Biophys Methods.

[14] Jones AC, Austin J, Hansen N, Hoogendoorn B, Oefner PJ, Cheadle JP (1999). Optimal temperature selection for mutation detection by denaturing HPLC and comparison to single-stranded conformation polymorphism and heteroduplex analysis. Clin Chem.

[15] Aboul-ela F, Koh D, Tinoco I Jr, Martin FH (1985). Base-base mismatches. Thermodynamics of double helix formation for dCA3XA3G + dCT3YT3G (X, Y = A,C,G,T). Nucleic Acids Res.

